# Head-Mounted Eye Tracking of a Chimpanzee under Naturalistic Conditions

**DOI:** 10.1371/journal.pone.0059785

**Published:** 2013-03-27

**Authors:** Fumihiro Kano, Masaki Tomonaga

**Affiliations:** 1 Primate Research Institute, Kyoto University, Inuyama, Aichi, Japan; 2 Japan Society for Promotion of Science, Chiyoda-ku, Tokyo, Japan; Lund University, Sweden

## Abstract

This study offers a new method for examining the bodily, manual, and eye movements of a chimpanzee at the micro-level. A female chimpanzee wore a lightweight head-mounted eye tracker (60 Hz) on her head while engaging in daily interactions with the human experimenter. The eye tracker recorded her eye movements accurately while the chimpanzee freely moved her head, hands, and body. Three video cameras recorded the bodily and manual movements of the chimpanzee from multiple angles. We examined how the chimpanzee viewed the experimenter in this interactive setting and how the eye movements were related to the ongoing interactive contexts and actions. We prepared two experimentally defined contexts in each session: a face-to-face greeting phase upon the appearance of the experimenter in the experimental room, and a subsequent face-to-face task phase that included manual gestures and fruit rewards. Overall, the general viewing pattern of the chimpanzee, measured in terms of duration of individual fixations, length of individual saccades, and total viewing duration of the experimenter’s face/body, was very similar to that observed in previous eye-tracking studies that used non-interactive situations, despite the differences in the experimental settings. However, the chimpanzee viewed the experimenter and the scene objects differently depending on the ongoing context and actions. The chimpanzee viewed the experimenter’s face and body during the greeting phase, but viewed the experimenter’s face and hands as well as the fruit reward during the task phase. These differences can be explained by the differential bodily/manual actions produced by the chimpanzee and the experimenter during each experimental phase (i.e., greeting gestures, task cueing). Additionally, the chimpanzee’s viewing pattern varied depending on the identity of the experimenter (i.e., the chimpanzee’s prior experience with the experimenter). These methods and results offer new possibilities for examining the natural gaze behavior of chimpanzees.

## Introduction

Human and nonhuman primates rely primarily on vision to retrieve information from the outside world. To retrieve visual information, primates rely on their eyes, especially on the central foveae, which capture less than 2 degrees of the visual field [1]. Thus, primates must actively move their eyes to select only necessary information from the array of information that exists in a real-life environment. Where do they look in such an environment?

Eye tracking is a technique that accurately measures these eye movements. In nonhuman primates such as macaques, a magnetic search coil method is commonly used for eye tracking [2]–[7]. However, this method requires the coil to be implanted on the eye surface of the subjects and the heads of the subjects to be firmly fixed in place by a chin rest or a bite bar. Thus, due to both ethical and physical constraints, this method is not applied to large primates such as great apes. A recent study solved this problem using a video-based, table-mounted eye tracker, allowing eye tracking without head restraints [8]. This eye tracker uses wide-angle camera lenses to search for both corneal and pupil reflections from the eyes and compensates for head movements (indicated by the corneal reflection) when calculating eye movement (indicated by the pupil reflection). This same method is commonly used in human infants [9] and, more recently, in dogs [10], [11].

However, despite its usability, the table-mounted eye-tracking method has an essential limitation. The experimental stimuli, typically 2D images or movies presented on a computer screen, are presented within the visual field of subjects, who are not able to interact with those stimuli. Thus, this method fails to capture the interactive nature of eye movements in a real-life environment. In human adults, video-based, head-mounted (i.e., wearable) eye trackers are used to examine the eye movements of subjects who are freely moving and interacting with real-life environments [12]. Previous studies have examined eye movements while participants executed various manual tasks, including making tea [13], making a sandwich [14], washing their hands [15], playing cricket [16], walking [17], and driving [18]. These studies have found that subjects fixate only on areas relevant to the task and do so only at the time at which relevant information is required. That is, the eye movements of subjects are goal directed and strictly dependent on the interactive context and the subjects’ actions.

The same head-mounted eye-tracking method has not been fully developed with nonhuman primates, and thus their goal-directed or natural eye movements are largely unexplored. One study was conducted on lemurs while they moved freely in a cage with conspecifics [19], [20]. The lemurs followed the gaze of their conspecifics in this real-life environment and showed differential eye-movement patterns when walking compared with being stationary. This head-mounted eye-tracking method has also been used with human infants [21] and with dogs [22] but not with phylogenetically closer animals (i.e., great apes).

In this study, we aimed to extend this eye-tracking method to a chimpanzee under naturalistic conditions. First, we evaluated the utility, reliability, and limitations of our method. Second, we examined whether our data on general patterns of eye movements (i.e., duration of individual fixations, length of individual saccades, etc.,) were comparable to the results reported in previous studies relying on table-mounted eye tracking (viewing still images) [8], [23]. Third, we examined how a real-life environment and the chimpanzee’s interaction with that environment affected the chimpanzee’s eye-movement patterns. Finally, we explored how the chimpanzee viewed the social stimuli (i.e. the experimenter’s face and body) under this interactive situation.

In our experiment we used a daily interactive situation that enhanced face-to-face communication between the chimpanzee and the human experimenter. We devised two experimental settings to alter the quality of the interaction between the subject and experimenter: a face-to-face greeting between the experimenter and the chimpanzee occurring when the experimenter initially appears in the experimental room (“greeting phase”) and subsequent task-related interaction involving both the experimenter’s and the chimpanzee’s manual gestures as well as fruit rewards (“task phase”).

Based on previous head-mounted eye-tracking studies with humans, we expected that the chimpanzee would view the body parts and scene areas most relevant to the ongoing context and actions. That is, during the greeting phase, we expected that the chimpanzee would view the experimenter’s face and body. In the task phase, we expected that the chimpanzee would view task-relevant areas, such as the experimenter’s gestures and the fruit reward.

With respect to the chimpanzee’s pattern of viewing the experimenter’s face and body, of particular interest was the pattern of viewing the face. Faces contain a rich store of information vital to their social lives, such as identity, emotion, and gaze direction [24]–[26]. The previous table-mounted eye-tracking studies found that chimpanzees primarily viewed the face and eyes when exposed to conspecific and human images [8], [27]–[29]. However, prolonged viewing of the face and eyes is less common in chimpanzees than in human subjects. Instead, chimpanzees view the body and mouth more frequently than do humans. These findings may reflect chimpanzees’ habitual communicative style or their limited use of long-bout facial communication, including making eye contact and reading subtle eye expressions and gaze directions. However, previous studies did not include interactions between subjects and stimuli, and thus subjects may have been less motivated to view faces and eyes. Additionally, because the experimental settings (free observation of images) and the presentation duration of stimuli (several seconds) in the previous studies were limited, the manner in which subjects alter the pattern of face viewing as a function of context and time remains unclear. In this study, we examined the extent to which the chimpanzee would view the experimenter’s face in the interactive situation. We then explored how context and actions would modify the chimpanzee’s pattern of viewing faces.

Additionally, as the quality of interaction depends on a chimpanzee’s relationship to an experimenter, we examined how the chimpanzee altered her viewing pattern depending on the identity of the experimenter. Although the previous table-mounted studies (using still pictures) examined chimpanzees’ viewing patterns for familiar and unfamiliar individuals, they failed to find differential viewing patterns. However, as mentioned above, their results may have been due to the lack of interaction between the chimpanzees and stimuli and to the short presentation duration of stimuli. We thus re-examined this issue in a real-life setting and expected to observe the chimpanzee’s novelty response (longer viewing) upon encountering the unfamiliar experimenter.

## Methods

### Subject

Pan, a female chimpanzee, aged 27 years old, participated in this study. Pan was a member of a social group comprising 13 individuals living in an enriched environment with a 700-m^2^ outdoor compound and an attached indoor residence [30]. The outdoor compound was equipped with climbing frames, small streams, and various species of trees. Access to the outdoor compound was available to Pan every other day during the day. Daily meals included a wide variety of fresh fruits and vegetables fed throughout the day supplemented with nutritionally balanced biscuits (fed twice daily) and water available *ad libitum*. Pan has been reared by humans and has experienced various cognitive experiments since youth [31]–[34]. The care and use of Pan adhered to the 2002 version of the Guidelines for the Care and Use of Laboratory Primates by the Primate Research Institute, Kyoto University. This experimental protocol was approved by the Animal Welfare and Care Committee of the same institute (no. 2010-023). For the daily experiments, Pan left her social group voluntarily on the request of experimenters, moved into the experimental booth with the guidance of experimenters, and moved back to her social group after the completion of experiments (approx. 1 hour).

### Monitoring Eye Movements

**Figure 1:**
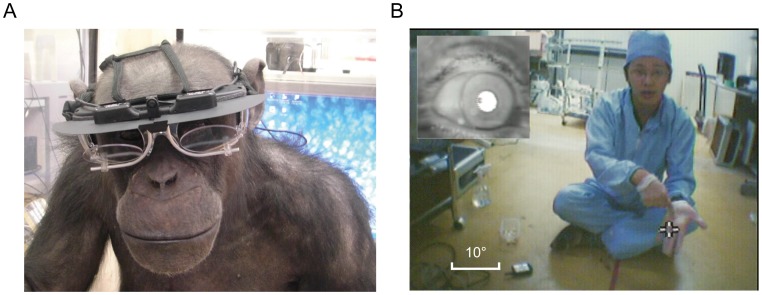
Experimental apparatuses. The eye tracker on Pan’s head (A) and the eye (left top) and scene-camera image (B). Cross mark indicates point-of-regard (POR). Also, see Video S2.

Pan’s eye movements were monitored using a commercial head-mounted eye tracker (Fig. 1A; 60 Hz, “Omniview”, ISCAN Inc., Woburn, MA, USA). This eye tracker has a temporal resolution of 60 Hz and a spatial resolution of <0.25° in recording the eye image (Fig. 1B). The accuracy of gaze-in-scene position (gaze position with respect to the world) was approximately 0.5° over a central 40° field when the calibration was accurate. Although this eye tracker is able to record both eyes of subjects, we abandoned the left-eye records of Pan due to the relatively lower position of her left eyelid (i.e., less robust to the eccentric eye movements compared with the right eye). Although tracking a single eye is known to cause a parallax error (i.e., a calibration error), especially in the distance of subject’s hand reach, this error was largely irrelevant to the current experiment, which did not include regions of interest in those areas.

The eye tracker was attached to goggles mounted on her head non-invasively (Fig. 1A). The goggles were fixed by four strings that passed along the top and sides of her head and were bound at the back of her head with a plastic clip. Due to the higher position of the ears in chimpanzees than in humans, the original temples of the goggles were replaced by wire temples shaped to fit Pan’s ears. Thus, the eye-tracker goggles were supported at multiple points on her head; her nose, the top and back of her head, and her ears. The goggles were immobile during the recording unless she touched them (data from these failed sessions were removed from the analysis; see below). Note that the goggles were fixed to Pan’s head only tightly enough to remain in place and could be removed by Pan herself any time in the session.

Two eye cameras were attached to the left- and right-top of the goggles, and they recorded the reflection of the eye image in a half mirror. Pupil and first Purkinje image centroids were extracted from the eye image, and eye-in-head position (the eye position with respect to the head) was calculated based on the vector difference between the two centroids. As this vector difference was independent of the absolute coordinates of the two centroids in the eye cameras, the eye-in-head position was robust to small movements of the goggles on the head. The scene camera was attached to the middle of the top of the goggles and provided a video recording (30 Hz) of the scene from her viewpoint (Fig. 1B; approx. 70×50° field in width and height). All data were stored in a small digital recorder, which was placed on the floor during recording.

Because Pan did not hesitate to wear the eye tracker, no habituation was necessary. However, to check the accuracy and increase the stability of recordings, we practiced the calibration procedure and conducted preliminary recordings with Pan for several weeks prior to the testing sessions.

### Calibration Procedure

**Figure 2:**
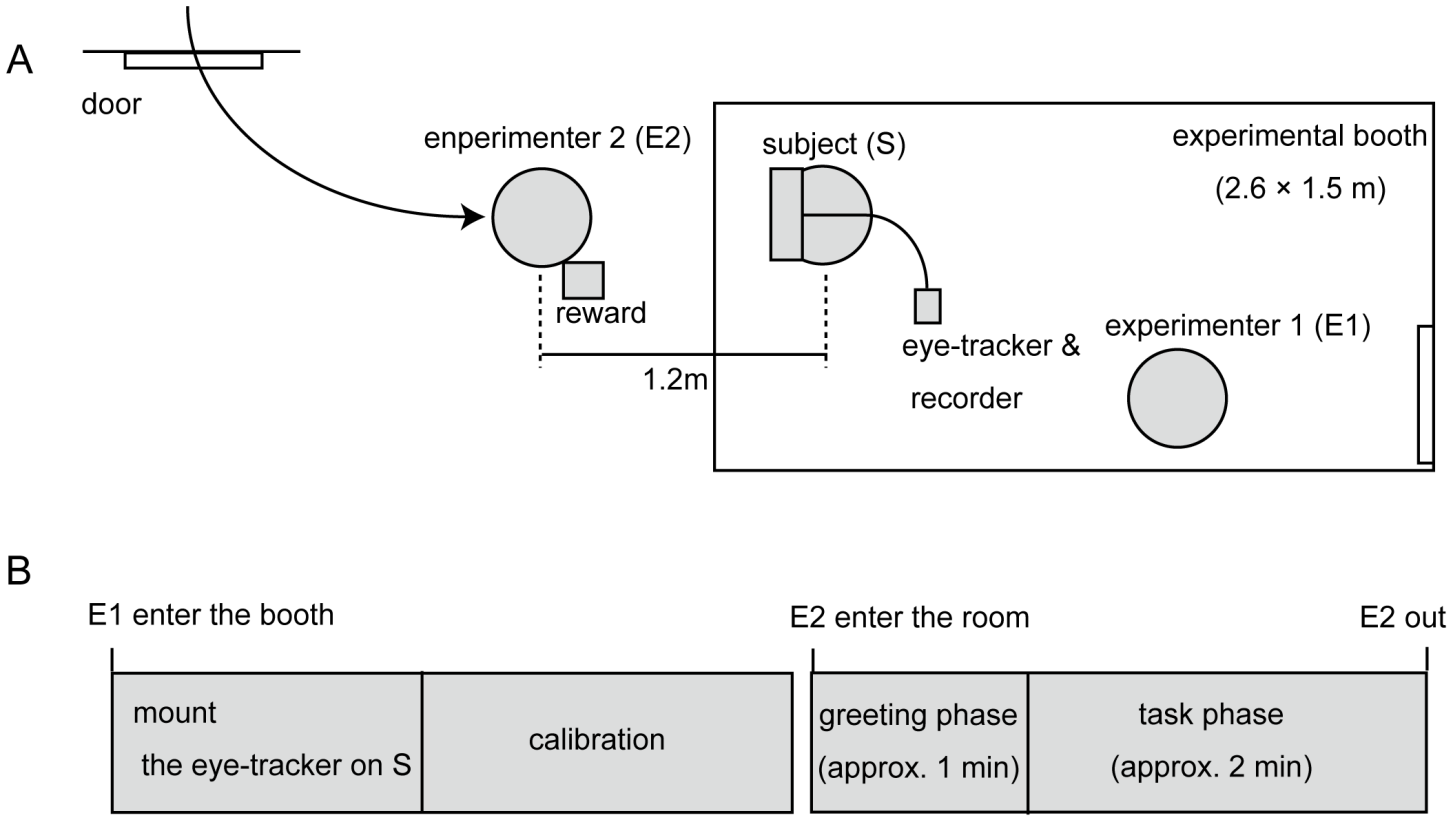
Experimental procedures. (A) Schematic of the experimental setting. (B) Time flow of an experimental session.

Two experimenters engaged in the calibration session. One remained inside an experimental booth (E1) and set the eye tracker on Pan’s head, and the other (E1′) remained outside of the booth and set the calibration frame. A five-point calibration was conducted each time before the daily session. The calibration points were set in a 58.5×51 cm frame, which was placed outside the booth at 1.5 m from Pan (22×19° in width and height). E1′ attracted her gaze to each calibration point several times by presenting small objects and rewards at that point (See Video S1). During the calibration procedure, Pan’s head was lightly held by E1, thereby preventing large head movements during calibration. Once these calibration procedures were finished, her head was set free, and E1 and E1′ moved away from her; E1′ moved completely away from her sight, and E1 remained in the booth but kept distance from her during the test session (Fig. 2a).

Off-line processing of the calibration data was performed on a PC by selecting the location of each calibration point on the scene-camera image and selecting the time at which Pan fixated on the point, as indicated by the eye-camera images (the eye movements that followed the objects/rewards). After processing the calibration data, the ISCAN system provided the point-of-regard (POR or eye-in-scene position), which was superimposed on the scene-camera image (cross mark in Fig. 1B). Failures in the calibration process were indicated by the loss of POR data on large sections of scene-camera images. The accuracy of the POR (the distance between the POR and the intended region of interest) was typically very small (within 1°) around the calibration surface (at the 1.5-m distance and within the central 40° field) but was larger when the POR was more distant from the surface. We thus conducted the main recordings around that area (see below). As a result, accuracy was around 0–2° during the recordings when estimated based on Pan’s eye movements following small rewards/objects (see Video S1).

### Testing Procedure

Experimenter 2 (E2) entered the room, sat on the floor in front of Pan, and gazed at, talked to, and gestured to her (Fig. 2A, 2B; “greeting phase”). After approximately 1 minute, E2 began the gesture task on which Pan had been trained for several years. This task lasted for approximately 2 minutes (“task phase”). During this task, E2 showed one of three actions to Pan, touching the nose, touching the palm, or clapping the hands, and gave the fruit reward (pieces of apple) when Pan reproduced that action. The fruit rewards were placed in a transparent box next to the experimenter, and the box and rewards were present at the fixed place throughout the entire session (i.e., during both greeting and task phases).

Six experimenters played the role of E2 across 2 days (total of 12 sessions). The six experimenters differed from one another in the quality of daily interactions with Pan. Four had been interacting with Pan on a daily basis (familiar), and the other two met her for the first time during the experiment (unfamiliar). Of the familiar four, two interacted daily with Pan in the room where this experiment was conducted (regular), but the other two interacted daily with her elsewhere, such as the chimpanzee residential area or another experimental room (irregular). That is, it was unusual for Pan to see the irregular E2 in the room where this experiment was conducted. The types of experimenters were thus termed “regular/familiar,” “irregular/familiar,” and “irregular/unfamiliar”. The order in which the different types of experimenter appeared within each session was randomized within the 12 sessions.

The entire procedure, including mounting the eye tracker on Pan and conducting the calibration and testing, lasted for 20–30 minutes. The sessions were terminated if Pan showed any signs of distress or if she touched the eye tracker. Sessions in which calibrations failed were eliminated from the analysis and repeated on another day (in total, 14 sessions, including two failed sessions, were conducted).

### Eye-movement Analysis

Lost eye signals (pupil or first Purkinje image) occurring as a result of blinks or downcast gazes amounted to 24% of the total recording time. Fixation was detected off-line based on the instantaneous velocity of the gaze-in-scene position. This velocity was calculated from the gaze-in-scene vector as a combination of the head and eye-in-head vectors. The head vector was calculated by tracking the coordinates of any object in the scene-camera images, and the eye-in-head vector was calculated from the POR coordinates given by the ISCAN system. A fixation (or smooth pursuit) was defined when the gaze-in-scene velocity was <30°/s. A saccade (velocity >30°/s.) shorter than 20 ms (i.e., one sample) and a fixation shorter than 50 ms (i.e., fewer than three samples) were regarded as noise and were integrated into the surrounding fixation and saccade, respectively, in that order. These criteria were chosen to match the POR movements projected on the scene-camera images. All analyses were conducted using MATLAB (www.mathworks.co.jp).

The following dependent variables were analyzed to represent the characteristics of Pan’s eye movements. 1) Viewing time: sum of all fixation durations (ms). 2) Fixation duration: duration of individual fixations (ms). 3) Fixation number: number of fixations. 4) Saccade length: length of individual saccades (degree).

### Coding of the Fixation Target and Actions

The fixation target was manually coded by inspecting the scene-camera image. The scene was divided into E2’s face (above the neck), hands (from the wrist), feet (from the ankle), and other body parts; the reward; and other areas (Fig. 2A). Inter-coder reliability was checked by another coder naïve to the experimental hypothesis using part of the coding footage (3 min.) and was categorized as excellent (Cohen’s *Kappa* = 0.84). The actions of E2 and Pan were recorded by three fixed cameras (SONY Handicam, www.sony.jp) aimed at E2 and at Pan’s front and back and coded by inspecting the footage on a frame-by-frame basis. The gaze data (shown in the scene-camera image) and action data (shown in the fixed-camera images) were temporally matched by inspecting the onset and offset of any actions appearing in both images.

## Results

**Figure 3:**
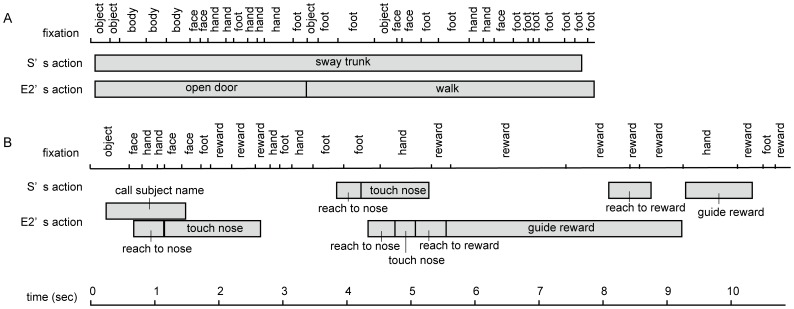
Examples of Pan’s fixations and Pan’s and E2’s actions as a function of time (s) during (A) greeting and (B) task phases. The ticks on the fixation axis indicate the beginning of each fixation.

Overall, our method enabled us to record Pan’s eye movements over the course of 3 minutes while Pan engaged in her usual interactions with E2; for example, Pan engaged in overt greeting gestures when E2 appeared (pant-grunting or swaying in five of the 12 sessions), and she performed the task actions and occasionally requested the reward after the task began (Fig. 3 and Table 1). During the task phase (approx. 2 min.), E2 produced the task actions an average of 13.6 times (*SD*: 4.1), and Pan reproduced the actions and obtained the reward an average of 9.9 times (*SD*: 3.1).

**Table 1:** Actions observed during the experiments.

Chimpanzee	
sway trunk (greeting gesture)	3
pant grunt (greeting gesture)	2
reach hand (request gesture)	89
short vocalization (request gesture)	49
clap hand	150
touch nose	99
tap palm	73
reach for reward	130
guide reward	119
self-scratch	73
Human interactive partners	
open door	24
walk	24
sit, stand up	24
call subject’s name	79
clap hand	45
touch nose	85
tap palm	34
reach for reward	125
guide reward	124

**Figure 4:**
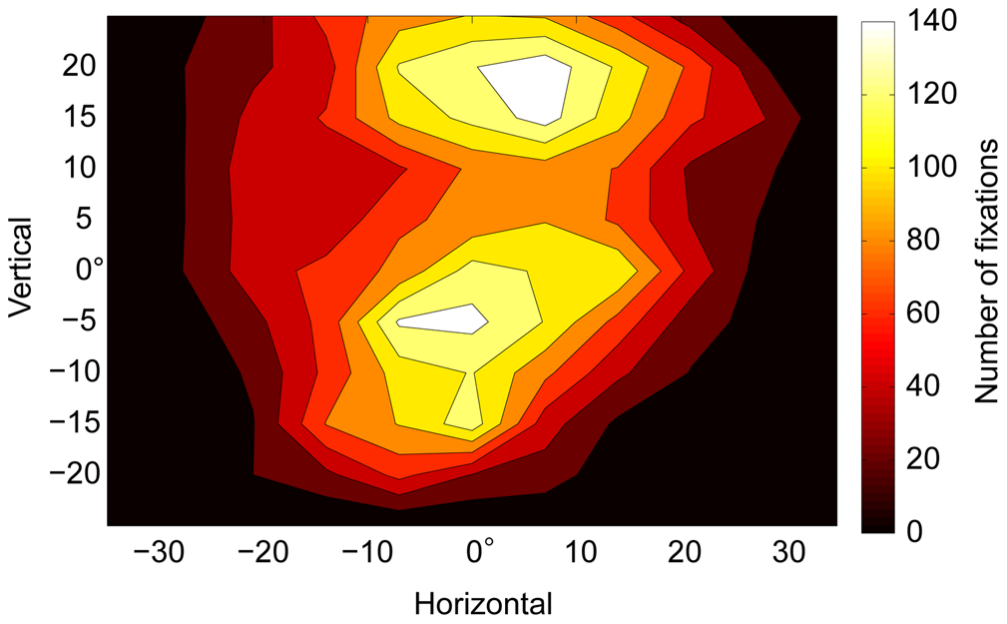
Spatial distribution of fixations on scene-camera images (i.e., eye-in-head).

With respect to the basic patterns of Pan’s eye movements (over all sessions), the average velocities of Pan’s head, eye-in-head, and gaze-in-scene movements were 11.9° (*SD*: 25.9), 56.1° (*SD*: 126.6), and 57.5° (*SD*: 130.5), respectively. This pattern indicates that Pan used eye movements more commonly than she used head movements to shift her gaze. The spatial distribution of Pan’s fixations on scene-camera images (i.e., eye-in-head) was shown in Figure 4. In general, her fixations were clustered in the middle of the horizontal axis and were more widely distributed along the vertical axis. This pattern indicates that Pan used head movements more frequently when shifting her gaze horizontally than when shifting her gaze vertically. The two peaks along the vertical axis largely correspond to the density of objects or body parts in the scene (i.e., a lack of interesting objects or body parts in the middle of the vertical axis; see Video S2).

**Figure 5:**
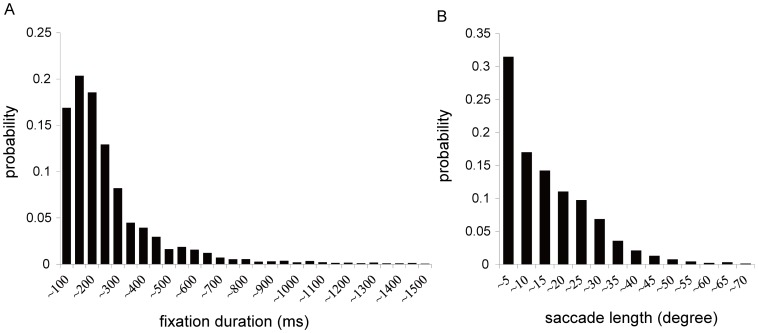
Probability distribution of (A) fixation duration (ms) and (B) saccade length (degree).

The temporal distributions of fixation durations (Figure 5A) and saccade lengths (Figure 5B) was similar to those in the previous reports about chimpanzees and humans [13], [17], [23], [35], [36]. That is, she exhibited a skewed distribution with a long right tail for the fixation duration and saccade length, with modes around 200–300 ms and 1–5 degrees, respectively. Table 2 shows the mean fixation duration and saccade length during each experimental phase. To examine changes in these variables across experimental phases, we conducted *t*-tests (total of 12 sessions) for each variable. We found no significant changes in either variable across the phases (fixation duration: *t*(11) = 1.60, *P* = 0.13; saccade length: *t*(11) = 0.62, *P* = 0.54).

**Table 2:** Fixation duration (ms) and saccade length (degree) during each experimental phase.

Experimental phase	Greeting	Task	Overall
Fixation duration (s.e.)	235 (10.2)	213 (9.4)	224 (7.0)
Saccade length (s.e.)	13.7 (0.53)	14.1 (0.37)	13.9 (0.33)

**Figure 6:**
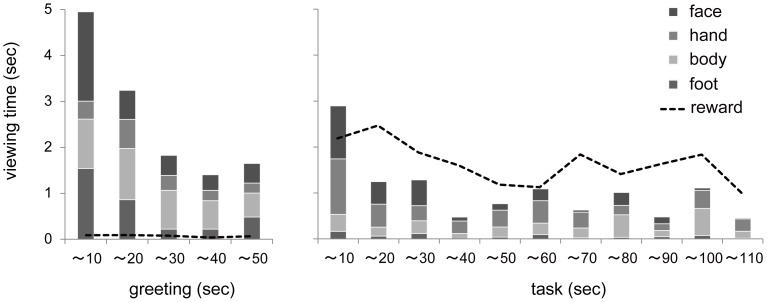
Viewing time (ms) for each scene area as a function of time during (A) greeting and (B) task phases.

Pan altered the viewing time for each scene area as a function of experimental phase and time (10-s time windows; Figure 6). In general, Pan decreased the time spent viewing E2 over the course of the greeting phase, increased it again when the task began, and decreased it again as time passed. Pan viewed the reward rarely (<1%) during the greeting phase, whereas she viewed it intensely during the task phase (>30%). To examine Pan’s viewing patterns for each of E2’s body parts according to the experimental phase, we compared the two phases (first 50 s) using a repeated-measures analysis of variance (ANOVA) with phase (2) and body parts (4) as factors (total of 12 sessions) and found a significant interaction between the two factors (*F*(3, 9) = 5.75, *P* = 0.018, *η* ^2^ = 0.65). The additional analyses showed a significant effect of body part during both greeting (*F*(3, 9) = 3.93, *P* = 0.048, *η* ^2^ = 0.56) and task (*F*(3, 9) = 4.04, *P* = 0.045, *η* ^2^ = 0.57) phases. Specifically, Pan viewed E2’s face, feet, and other body parts for a particularly long time during the greeting phase and viewed E2’s face and hands for a particularly long time during the task phase.

**Figure 7:**
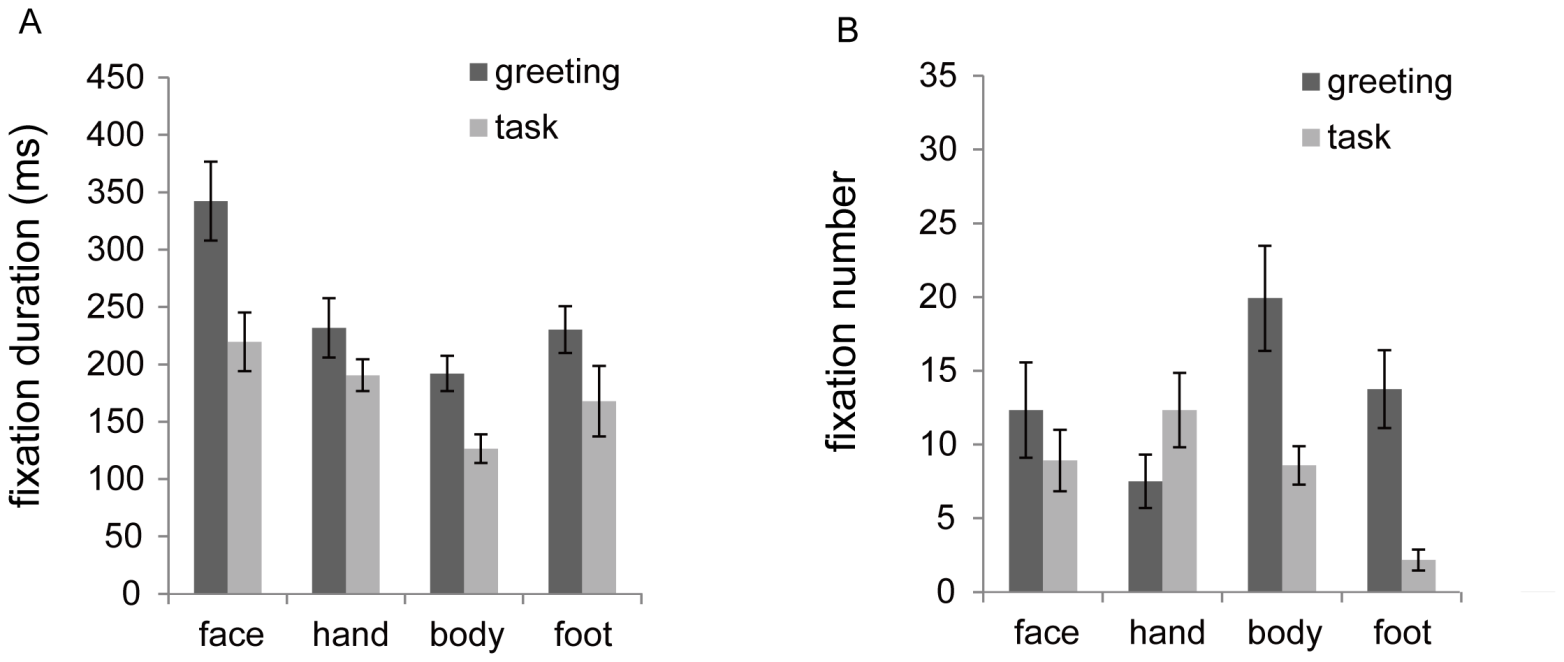
Average of (A) fixation duration (ms) and (B) saccade length (degree) for each scene area during the greeting and task phases (first 50 s).

Pan also altered the number and duration of fixations on each of E2’s body parts as a function of experimental phase (note that *viewing time = fixation duration*×*fixation number*; Figure 7). To examine changes in these parameters across experimental phases, we conducted a repeated-measures ANOVA with phase (2) and body parts (4) as factors (total of 12 sessions). As with viewing time, we found a significant interaction between the two factors with respect to number of fixations (*F*(3, 9) = 7.90, *P* = 0.007, *η* ^2^ = 0.72), whereas no significant interaction was found with respect to fixation duration (*F*(3, 4) = 0.34, *P* = 0.79, *η* ^2^ = 0.20; note that these data include null values due to no fixation to particular body parts in a few sessions). In terms of fixation duration, we found significant main effects of phase (*F*(1, 6) = 8.26, *P* = 0.028, *η* ^2^ = 0.57) and body parts (*F*(3, 4) = 10.83, *P* = 0.022, *η* ^2^ = 0.89), indicating that Pan exhibited longer fixation durations during the greeting than during the task phase and longer fixation durations for the face than for other body parts during both experimental phases.

Pan’s viewing pattern for E2 was related to her own and to E2’s actions. Pan offered greeting gestures to E2 five times during the greeting phase (Table 1). While performing these gestures, Pan fixated on E2 exclusively (90% of all fixations), especially on E2’s face and feet (43% and 33% on the face and feet, respectively). Additionally, Pan fixated primarily on E2’s feet when E2 was walking (80% of all fixations on E2, 40% on E2’s feet; 14% on E2’s face). During the task phase, Pan fixated primarily on the face and hands when those parts were cued by E2 during task actions (i.e., touching the nose, clapping the hands, and touching the palm; 59% and 41% of all fixations were targeted to the face and hands, respectively).

Pan exhibited anticipatory fixation on E2’s actions, particularly reaching for the reward (i.e., fixation on the reward before E2 grasped it; 84% of all reaching actions). On those occasions, Pan fixated the reward an average of 313 ms (*SD*: 146 ms; median: 308 ms) before E2 grasped the reward, and E2’s reaching action lasted an average of 666 ms (i.e., from the onset of the hand movements until the onset of the grasping movements; *SD*: 114 ms; median: 658 ms). These anticipatory fixations were likely triggered by E2’s hand actions rather than by E2’s head-gaze moving toward the reward or by the other task cues because just before those anticipatory fixations, Pan did not fixate on the face (1% of all fixations) but rather was looking elsewhere, and these anticipatory fixations were initiated after the onset of E2’s reaching action (99% of all anticipatory fixations).

**Figure 8:**
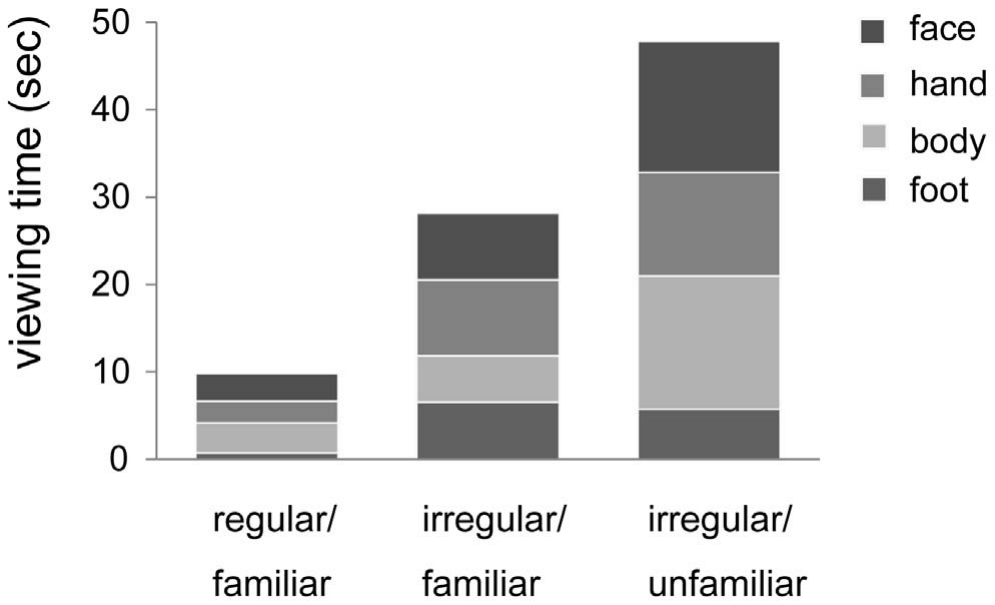
Viewing time for E2 as a function of familiarity with E2 during the greeting phase (first 50 s). Error bars indicate standard errors. *p<0.05.

The familiarity status of E2s affected Pan’s viewing time for E2 during the greeting phase (first 50 s; Figure 8). We conducted a repeated-measures ANOVA with familiarity (3) and body parts (4) as factors (four sessions for each familiarity factor; total of 12 sessions). We found significant main effects for familiarity (*F*(2, 9) = 4.91, *P* = 0.036, *η* ^2^ = 0.52) and body parts (*F*(3, 7) = 6.43, *P* = 0.020, *η* ^2^ = 0.73), indicating that Pan viewed regular/familiar E2s least strongly, irregular/unfamiliar E2s (especially the face and foot) most strongly, and irregular/familiar E2s at an intermediate level.

## Discussion

Pan wore the lightweight eye tracker on her head for more than 3 minutes in each session, and no physical restraints were necessary. This recording duration is far longer than those in the previous studies that presented still images to chimpanzees (2–3 s) [8], [23]. The eye tracker did not seem to inhibit her head movements in that she moved her head frequently (see Video S2). Additionally, the eye tracker did not seem to inhibit her bodily/manual movements given that she also exhibited a wide range of her usual behavioral repertoire (Table 1). One clear limitation of this study is that we obtained the data from a single chimpanzee due to the constraints of the experimental setting (i.e., one experimenter remains with the chimpanzee in the same experimental booth). In the future, we aim to habituate or find more subjects.

Despite this limitation, the results of this study included several interesting findings that allow us to re-evaluate previous table-mounted eye-tracking studies of chimpanzees. First, the general patterns of eye movements, in terms of fixation duration and saccade length were very similar to those reported by previous studies [8], [23]. That is, the data for both fixation duration and saccade length were characterized by distributions with long right tails. These skewed distributions, demonstrating the variability of Pan’s eye movements, suggest that the she flexibly controlled fixations and saccades, as has been demonstrated in previous studies with humans [13], [36], [37]. It should also be noted that the average fixation duration (254 ms) was close to the value observed in previous studies (∼250 ms) [8], [23], [28].

Second, the general patterns of Pan’s face viewing were similar to those reported by previous table-mounted eye-tracking studies [8] despite the fact that different experimental settings were adopted in each study. That is, Pan viewed the face for a longer period of time than she viewed the other body parts, and she demonstrated longer fixations on the face than on other body parts (Fig. 6A). However, long-bout face viewing, which has been commonly reported among humans in previous studies (long duration of fixations >500 ms or successive fixations on the faces), was not frequently observed in this study. Instead, Pan typically alternated her gaze between the face and the other body parts (e.g., feet and hands) when attending to the experimenter.

However, these results do not suggest that the interactive contexts did not play a role in Pan’s viewing patterns. Indeed, Pan flexibly modified her viewing patterns for faces and other scene areas depending on the ongoing context or action. For example, Pan rarely viewed the fruit rewards and instead viewed the experimenter during the greeting phase, although she focused on the fruit rewards after task initiation. Additionally, Pan concentrated on the face and feet during the greeting phase and on the face and hands during the task phase. These differences can be explained by the differential bodily/manual actions produced by Pan and the experimenter during each experimental phase. That is, Pan viewed the face and feet when she was performing greeting gestures (e.g., pant grunting, swaying) directed toward the experimenter, and Pan viewed the face and hands when the experimenter was gesturing toward her in the task phase (e.g., touching the nose and hands). Thus, overall, although Pan viewed the experimenter when she and the experimenter were engaged in actions directed toward each other, on those occasions, Pan viewed the experimenter’s whole body (e.g., feet, hands) and not necessarily the face. Future studies should examine chimpanzees’ viewing patterns in situations that facilitate production of a wider variety of actions, such as interactions with conspecifics, to further clarify chimpanzees’ habitual communicative styles.

In this study, we also found that Pan’s viewing pattern was dependent on her prior experiences with the experimenter (i.e., familiar/unfamiliar, regular/irregular) during the greeting phase. This finding is particularly interesting given that previous studies that presented images of familiar and unfamiliar people to chimpanzees (including this subject) did not find any significant effect of familiarity [8]. This difference between studies may be explained in terms of habituation speed. That is, this study observed the effect of familiarity 10 seconds after the appearance of the experimenter. However, the previous study presented the images for only a short duration (3 s.). This suggests the importance of using extended time scales to examine chimpanzees’ viewing response to social stimuli.

Apart from the pattern of face/body viewing, the method employed in this study can be used for other research purposes in the future. We suggest two directions for future research. The first is interspecies comparisons with humans with respect to basic eye-movement controls. It remains unclear how chimpanzees differ from humans in the duration of individual fixations, the length of individual saccades (Fig. 5), and the use of head and eye movements in shifting gaze (Fig. 4) in a real-life environment. In this study, the chimpanzee did not alter her overall pattern of fixation duration and saccade length depending on context (Table 2). This is consistent with the results of some studies conducted with human adults [38] but not with those of others [39]. Additionally, previous studies have found that chimpanzees engage in shorter fixations and longer saccades than do humans when scanning scenes (i.e., chimpanzees scanned scenes more quickly and more widely than did humans). It is unclear how this finding applies to real-life situations. Furthermore, due to the limited contexts and lack of actions in previous studies, the functions involved in shorter/longer duration of fixations of each species remain unclear.

The second direction for future research relates to gaze following and anticipatory looking in chimpanzees. A number of behavioral studies have been conducted on how monkeys and great apes use experimenter-given social directional cues such as gazing, pointing, and reaching for an object in a choice task [34], [40]–[44]. Although monkeys and great apes are able to use these directional cues in a task, they are limited in their ability to use gaze cues, especially eye-only cues (no head direction). However, these previous studies did not clarify how subjects looked at the experimenter’s actions with anticipation. In this study, the chimpanzee frequently (more than 80% of all occasions) looked at the experimenter’s reaching action with anticipation, but she did not show clear evidence of following the experimenter’s gaze. Further studies are necessary to examine this issue more thoroughly.

In conclusion, this study offers a new method for examining the bodily, manual, and eye movements of a chimpanzee at the micro-level while the chimpanzee interacts with a real-life environment. We found that the general viewing patterns, such as the duration of individual fixations, the length of individual saccades, and the pattern of face viewing, were similar to those reported by previous eye-tracking studies despite differences in experimental settings. However, ongoing context and actions were critically related to the chimpanzee’s eye movements. These methods and results offer new possibilities for examining the natural gaze behavior of chimpanzees.

## Supporting Information

Video S1 Calibration procedure. Cross mark indicates point-of-regard (POR) after the calibration procedure, which suggests the accuracy of POR.
(WMV)

Video S2 Scene-camera image with point-of-regard (POR; cross mark). The first half of image was recorded during the greeting phase, and the latter half was recorded during the task phase.
(WMV)
